# Loneliness Mediates the Link Between Indirect Self-Destructive Behavior and Life Satisfaction in Women from Dysfunctional Family Backgrounds

**DOI:** 10.3390/brainsci15121344

**Published:** 2025-12-18

**Authors:** Małgorzata Szcześniak, Martyna Słotwińska, Wojciech Rodzeń, Rafał Pietruszka

**Affiliations:** 1 Faculty of Social Sciences, Institute of Psychology, University of Szczecin, 71-017 Szczecin, Polandwojciech.rodzen@usz.edu.pl (W.R.); 2 Faculty of Social Sciences, The Pontifical University of John Paul II, 31-002 Cracow, Poland

**Keywords:** indirect self-destructive behavior, life satisfaction, loneliness, women, dysfunctional family

## Abstract

**Background:** Among individuals who perceive their families of origin as dysfunctional, both indirect self-destructive behaviors and loneliness seem to be important correlates of life satisfaction. However, a review of the existing literature reveals a notable absence of studies that examine these variables within a single analytical framework. **Objectives**: Given the limited number of studies examining the relationship between indirect self-destructive behavior, loneliness, and life satisfaction among women from dysfunctional family backgrounds, this study aimed to: (1) examine the association between indirect self-destructive behavior and life satisfaction among women experiencing different types of family dysfunction, and (2) determine whether loneliness mediates this relationship. **Methods**: The research was conducted among 207 women aged 18 to 63 (*M* = 30.78 years; *SD* = 9.945) who were raised in dysfunctional families. The Indirect Self-Destructiveness Scale [ISDS-25], the Satisfaction with Life Scale [SWLS], and the De Jong Gierveld Loneliness Scale [DJGLS] were used. **Results**: Statistically significant correlations were obtained between all pairs of variables: (1) loneliness and life satisfaction (*r* = −0.50 ***); (2) life satisfaction and indirect self-destructive behavior (*r* = −0.31 ***); (3) loneliness and indirect self-destructive behavior (*r* = 0.20 **). Moreover, mediation analyses showed two outcomes. First, loneliness acted as a mediator in the relationship between indirect self-destructive behavior and life satisfaction. Second, indirect self-destructive behavior had a mediation effect on the relationship between loneliness and life satisfaction. **Conclusions**: The conducted study and the obtained results fill a significant gap in the knowledge about indirect self-destructive behaviors, loneliness, and their relationship with the dysfunction of the family of origin. Thus, they constitute a new resource of expertise for interdisciplinary teams working with adults.

## 1. Introduction

A dysfunctional family, also referred to as a disharmonious family [1] or a risky family [2], is commonly defined as one whose relational patterns undermine or even prevent proper functioning [3] rather than support the emotional and physical well-being of its members [4]. Such families are characterized by increased [5] and chronic conflict [6], instability [2], excessive parental control [5], perfectionistic expectations [7], limited empathy, emotional coldness [3], heightened criticism [8], a diminished sense of safety [9] and security [10], difficulties managing stress and poor problem-solving strategies [11]. Dysfunctional family identity involves ineffective [11], restricted or impaired communication [12], often expressed through relational distance, emotional withdrawal, or complete cutoff [13]. Dysfunction in the family of origins is related to alcoholism and mental illness [6], intimate partner physical and sexual violence [2], anxiety [11] and depressive symptoms [6].

In view of the far-reaching impact that dysfunctional family has on psychological adjustment, it is reasonable to expect that it also shapes broader indicators of well-being, including life satisfaction. In fact, findings from empirical studies demonstrate that psychological well-being [14,15], self-fulfillment [16], and the ability to reframe negative experiences into resilient outcomes [17] depends on family functioning [18]. People with experience of healthy family express higher levels of life satisfaction [19]. Conversely, living in a dysfunctional family can seriously compromise the well-being of its members [18].

Among the various factors associated with life satisfaction in individuals who perceive their families of origin as dysfunctional, both indirect self-destructive behaviors and the experience of loneliness appear to play an important role. A review of existing research indicates that studies integrating these variables within a single analytical framework are notably lacking. Moreover, relatively few investigations focus specifically on adult women (much more research concerns children and adolescents), despite evidence suggesting that indirect self-destructiveness may manifest differently across genders [20,21,22].

Given the limited empirical attention to the interplay between indirect self-destructive behavior, loneliness, and life satisfaction in this population, the present study sought to: (1) examine the association between indirect self-destructive behavior and life satisfaction among women reporting different forms of family dysfunction; (2) determine whether loneliness mediates this relationship; and (3) examine whether an alternative mediation model is plausible in the context of life satisfaction, that is, whether loneliness may function as the explanatory variable and indirect self-destructive behavior as the mediator.

### 1.1. Indirect Self-Destructive Behavior

While the concept of self-destructiveness is typically associated with direct behaviors such as substance abuse, suicide attempts, or self-harm [23], the 1980s marked the distinction of a form characterized by multiple, often common, repetitive, and indirectly harmful behaviors [24,25]. Most authors conceptualize indirect self-destructiveness as a tendency to engage in behaviors that increase the likelihood of negative and reduce the likelihood of positive outcomes for the individual [25]. This less tangible and more difficult-to-recognize form is defined by chronicity, repetition, and indirectness, meaning that no explicit attack on one’s own body or life is present [26]. Instead, individuals are guided by emotional factors [25] and yield to hazardous impulses [27], leading to harm that manifests only after a delay. Consequently, indirect self-destructiveness is characterized by the extended time interval between action and consequence [26]. Although many such behaviors appear common or “normal”, it is their repetition, diversity, and co-occurrence that constitute important diagnostic markers.

According to Okasha [28] (p. 883), indirect self-destruction consists of “taking a life-threatening risk without the intention of dying, mostly repeatedly and often unconsciously.” Evidence suggests that persistent and unrecognized engagement in such behaviors may serve as a cry for help [29,30] and can lead to deterioration of mental or physical health, or even other destructive consequences [28], including premature death [31]. Individuals with high chronic self-destructiveness tend to experience limited social support [32], lower levels of life satisfaction and religious commitment [10], and heightened suicidal ideation [33].

Manifestations of indirect self-destructiveness include various forms of self-neglect [31], health-care negligence [26,27], intentional suffering [27], feelings of helplessness [34,35], self-defeat [36], risky or impulsive behaviors, yielding to temptations, and addictions [35,37]. Thus, indirect self-destructiveness encompasses both passive forms (e.g., neglecting one’s health) [26] and active forms (e.g., taking unnecessary risks), both of which can diminish or shorten quality of life [38]. As Kelley et al. [25] note, chronic self-destructiveness appears to function as a personality dimension influencing behavior across a broad range of contexts and stages of life.

Although precise prevalence estimates are lacking, studies show that indirect self-destructive behaviors occur in nonclinical populations [27,39] as well as a variety of clinical groups, including individuals with complicated grief [40], patients undergoing chronic hemodialysis [41], older adults and nursing home residents [29,42,43], and individuals with Buerger’s disease [44]. Moreover, such behaviors (e.g., non-suicidal self-injury, borderline impulsivity, acute grief) are common among individuals with histories of family dysfunction [45] and among those presenting preoccupied, dismissive [46], or disorganized attachment styles [47].

Research on indirect self-destructiveness includes both clinical and nonclinical populations, and the underlying behavioral mechanisms have been shown to manifest across contexts [48,49,50]. Clinical samples tend to exhibit more severe or health-critical forms of indirect self-destruction [51]. In contrast, studies of nonclinical groups indicate that such behaviors may arise in everyday functioning as responses to chronic stress, impaired emotion regulation, or adverse family environments [22,52]. Accordingly, although the present study focuses on a nonclinical sample of adult women who grew up in families with dysfunction, mechanisms identified in clinical research are treated as conceptual parallels that inform broader patterns of indirect self-destructive behavior rather than as direct clinical analogies.

### 1.2. Indirect Self-Destructive Behavior and Life Satisfaction

Life satisfaction, considered a crucial indicator of well-being [53], depends on personal traits and various aspects of life [54]. Among the many factors that affect the subjective and cognitive evaluation of one’s own life, a tendency toward behaviors that worsen outcomes seems essential. Results reported by empirical researchers suggest that engagement in intentional self-harmful actions can cause physical damages [55] and negatively affect the individual’s overall well-being [25,56,57,58]. People with a tendency toward self-destructive behaviors present higher levels of anxiety and lower self-esteem [59]. They are characterized by experiencing more interpersonal abuse and depression, as well as more externalizing attitudes and less control in relationships than people with low chronic self-destructiveness [60]. People with a self-destructive profile are characterized by lower life satisfaction and report lower psychological well-being [61,62]. It has been found that Finnish adolescents who deliberately harmed themselves also declared dissatisfaction with life [63]. Le et al. [64] show that participants who reported intentionally engaging in actions to cause pain or injury to themselves had lower well-being, life satisfaction, or overall health-related quality of life compared to those not engaging in self-harm.

Moreover, a coherent link between life satisfaction and indirect self-destructive behavior among women who grew up in families with dysfunction can be explained within stress-vulnerability models [65,66]. Early adverse family experiences, such as neglect, conflict, or psychological violence, constitute chronic stressors that shape maladaptive emotion regulation [67], reduce access to social support, and increase vulnerability to self-defeating behaviors [68]. In such conditions, indirect self-destructive actions may become a maladaptive strategy for coping with stress, while loneliness emerges as a consequence of disrupted relational patterns [69,70]. Because chronic loneliness further heightens stress sensitivity and lowers well-being, it can act as a key mechanism through which indirect self-destructiveness leads to reduced life satisfaction. This model provides a coherent theoretical rationale for the expected associations in this specific population.

### 1.3. Indirect Self-Destructive Behavior and Loneliness

Loneliness is a subjective, unwelcome experience resulting from deficiencies in social relationships [71,72]. The literature indicates that loneliness may function both as an independent variable affecting human functioning and as an outcome shaped by other factors. Previous research has highlighted its significant role in the emergence of self-destructive behaviors across developmental groups [73,74,75]. Individuals reporting higher levels of loneliness and a lack of social support are more likely to engage in self-harm [71,73], self-aggression [76], and negative self-evaluation [77], which aligns with the assumptions of the Interpersonal Theory of Suicide [78]. Consequently, social support and a sense of belonging serve as important protective factors [79] that improve overall quality of life.

Although no studies have examined the reverse association—namely, whether indirect self-destructive behaviors contribute to loneliness as an outcome—existing evidence suggests that the relationship between these constructs may be cyclical. Certain forms of self-destructiveness [80], such as chronic self-neglect [81], low self-regard [82], self-deprecation [83], and substance-related problems [80,84], can influence both the individual and their close environment, increasing the likelihood of perceived or anticipated rejection [82] and, consequently, vulnerability to loneliness. Self-harming thoughts and behaviors have been shown to predict later feelings of interpersonal disconnection, social withdrawal, and perceived rejection, suggesting that self-destructiveness may gradually erode relational functioning [85,86,87,88]. These findings support the view that indirect self-destructive behavior can initiate social–emotional processes characterized by shame, anticipatory rejection, and fear-based reactions from others, ultimately intensifying loneliness. Moreover, research demonstrates that insecure attachment is strongly associated with heightened loneliness across adulthood, as difficulties in trusting others and regulating relational distance diminish perceived social support [89,90]. Deficits in social skills—such as limited assertiveness, impaired emotional expression, or difficulties in initiating and maintaining social interactions—further heighten vulnerability to chronic loneliness [52]. Additionally, childhood adversity, including emotional neglect, inconsistent caregiving, and exposure to dysfunctional family dynamics, has been consistently linked to elevated loneliness in adult life [91,92]. These findings underscore that loneliness frequently emerges as a downstream consequence of early relational disruptions and interpersonal difficulties, mechanisms highly relevant to the present study’s focus on women raised in dysfunctional families.

### 1.4. Loneliness as a Mediator

While the direct relationship between indirect self-destructive behaviors and life satisfaction is empirically confirmed, little is known about the potential mediating role of loneliness in this association. To address this gap, we examined whether the unpleasant experience of loneliness acts as a key mechanism through which an individual’s tendency toward indirect self-destruction relates to their subjective evaluation of overall happiness. Because cross-sectional mediation requires theoretical and/or empirical justification of the temporal ordering of variables [93], our assumptions draw on studies that demonstrate analogous relational patterns.

Regarding the association between indirect self-destructive behaviors and loneliness, Wedajo et al. [94] found that maternal self-harm thoughts were significantly associated with loneliness. Other research has shown that feelings of inner emptiness can reduce life satisfaction by eliciting fear-based responses. This relational sequence aligns with the conceptualization of loneliness as both a construct shaped by other factors (e.g., manifestations of indirect self-destruction) and one that influences subsequent outcomes (e.g., life satisfaction). Indirect self-destructive behaviors—such as neglecting personal needs, engaging in risky interpersonal patterns, or chronically avoiding problem-solving—are associated with greater loneliness and social disconnection [48,50]. Individuals who repeatedly engage in such behaviors may erode their social resources, weaken relational bonds, and increasingly withdraw from supportive relationships, which contributes to heightened loneliness. Complementary evidence indicates that family dysfunction marked by inconsistent communication, emotional unavailability, or maladaptive conflict patterns predicts adult loneliness by undermining trust, attachment security, and relational self-efficacy [20,91]. Together, these findings support both a direct link between family dysfunction and loneliness and a potential pathway through elevated engagement in indirect self-destructive tendencies.

Most studies show that the absence of supportive social relationships is a risk factor for reduced well-being [95] and lower life satisfaction [96,97,98] across diverse age groups, cultures, and contexts [99]. Longitudinal research further strengthens this conclusion. For example, Martinez et al. [100] reported that steeper increases in loneliness during the COVID-19 pandemic corresponded to sharper declines in life satisfaction. Another panel study demonstrated that higher loneliness predicted lower life satisfaction over a 15-month period [101]. Additionally, extensive longitudinal evidence shows that elevated loneliness consistently predicts subsequent decreases in life satisfaction [97,102]. Taken together, these studies provide a compelling theoretical and empirical rationale for conceptualizing loneliness as the primary mediator linking indirect self-destructive behavior to diminished life satisfaction.

In light of the theoretical and empirical evidence discussed in the Introduction, we advanced the following hypotheses:

**H1.**  *Indirect self-destructive behavior is negatively associated with a cognitive judgment of women’s perceived quality of life as a whole.*

**H2.**  *Indirect self-destructive behavior is positively associated with women’s loneliness.*

**H3.**  *As the feeling of loneliness increases, the level of satisfaction decreases*.

**H4.**  *Indirect self-destructive behaviors are associated with life satisfaction via loneliness*.

**H5.**  *Loneliness is associated with life satisfaction via indirect self-destructive behaviors*.

The first mediation (H4) model is exploratory, as the existing literature provides limited theoretical justification and empirical evidence supporting the temporal ordering of indirect self-destructive behavior in relation to loneliness. In contrast, the second model (H5), well-grounded in theoretical frameworks and empirical research on the relationship between loneliness and indirect self-destructive behavior, was assumed to determine whether this association would be confirmed in our study.

## 2. Materials and Methods

### 2.1. Participants and Procedure

The research was conducted among 207 women aged 18 to 63 (*M* = 30.78 years; *SD* = 9.945) who were raised in dysfunctional families. We used an online survey created on the Google Forms platform (Google LLC, Mountain View, CA, USA). The survey included a brief introduction with instructions for the study, as well as questions regarding gender, age, place of residence, type of dysfunction experienced in the family of origin, and measurement tools. The majority of the study group lived in large cities with more than 500,000 inhabitants (33.8%; *N* = 70), followed by cities with 200,000 to 500,000 inhabitants (17.4%; *N* = 36), cities with 100,000 to 200,000 inhabitants (10.6%; *N* = 22), and cities with 20,000 to 100,000 inhabitants (23.2%; *N* = 48). 15% (*N* = 31) lived in villages. The data were obtained through a structured interview in which women were asked to indicate the type of family dysfunction that, in their opinion, was the most characteristic of the family in which they were raised. Respondents were allowed to choose only one category, specifically the one they felt most affected their functioning within the family. This approach was justified because different types of dysfunction often co-occur, and our aim was to determine which type each respondent perceived as having the strongest impact. Table 1 contains a detailed description of the type of dysfunction occurring in the family of origin of the surveyed women.

The group was purposefully selected from among the beneficiaries of associations, forums, and groups on the Facebook website, comprising individuals who were raised in dysfunctional families: “DDA-DDD—Adult Children of Alcoholics-Adult Children from Dysfunctional Families”; “DDA-DDD-SYNDROM”; “DDA/DDD GIRLS”. These groups bring together individuals who identify as adults raised in dysfunctional families and serve as communities offering mutual support. The sample also included participants affiliated with the “OD-DO” Association, which provides therapy for individuals from dysfunctional family backgrounds. After granting permission to participate in the study, the association’s administrators forwarded an invitation to complete the survey to them.

Participation in the project was voluntary and anonymous, and respondents were notified of this before starting the study. Their informed and written consent was obtained after participants were informed about the aim of the study, its anonymous character, and the lack of negative mental health effects on them. The present study was reviewed and approved by the Ethics Committee of the University of Szczecin (KB 53/2024, 5 December 2024) and conducted in accordance with the principles outlined in the Declaration of Helsinki.

### 2.2. Indirect Self-Destructiveness Scale

The Indirect Self-Destructiveness Scale [ISDS-25] [25], adapted by Pilarska and Suchańska [103], is a shortened version of the original Indirect Self-Destructiveness Scale. The ISDS consists of 25 items, which refer to transgressive and risky behaviors (7 items), health neglect (4 items), personal and social neglect (8 items), inattentiveness and carelessness (2 items), and passivity toward difficulties and failures (4 items). The subjects provide answers on a 5-point Likert scale, where A = strongly agree through E = strongly disagree. The result of the scale is the sum of all points. The higher the value of the sum, the greater the generalized tendency to indirect self-destructive behaviors. Despite assessing several distinct facets of indirect self-destructiveness, the authors of the Polish adaptation of the ISDS-25 argue that the scale has a one-dimensional structure, whereby the total score constitutes an index of generalized indirect self-destructiveness. In the original study, analysis of the psychometric properties of the short ISDS-25 indicated satisfactory reliability (between α = 0.80 and α = 0.88) and theoretical validity. In the current study, the reliability of the whole scale was very good with α = 0.81.

### 2.3. Satisfaction with Life Scale

The Satisfaction with Life Scale [SWLS] [104] in the Polish adaptation by Juczyński [105] is a scale consisting of 5 statements measuring the general index of life satisfaction. The examined person, referring to their previous experience, makes an assessment on a 7-point Likert scale, where 1 = I completely disagree, and 7 = I completely agree. The scale is the tool designed to examine adults regardless of their mental health, and the total score is the sum of the examined person’s assessments. The scale is characterized by satisfactory psychometric properties both in non-clinical (Cronbach’s α = 0.81) and clinical populations (Cronbach’s α = 0.91) [106]. In the present study, reliability was very high and amounted to α = 0.86.

### 2.4. De Jong Gierveld Loneliness Scale

The De Jong Gierveld Loneliness Scale [DJGLS] [107] in the Polish adaptation by Humenny and Grygiel [108] is a scale containing 11 statements measuring two dimensions of loneliness. The statements are divided into five positive and six negative items. The positive ones refer to the social dimension of loneliness, while the negative ones refer to the emotional dimension. Despite the presence of two dimensions in the scale, the tool is considered unidimensional. The participants respond using a 5-point Likert scale, where 1 = definitely yes, and 5 = definitely no. The overall result is the sum of the respondents’ responses and expresses the general degree of loneliness. The psychometric properties are satisfactory in general populations (Cronbach’s α = 0.89) and in clinical groups (Cronbach’s α = 0.85) [109]. In the current study, the reliability of the scale was very high, with α = 0.91 for the overall result, α = 0.85 for the social dimension, and α = 0.86 for the emotional dimension.

### 2.5. Statistical Analysis

All analyses were performed using IBM SPSS Statistics version 27.0 (IBM Corp., Armonk, NY, USA). The mediation model was analyzed with Andrew Hayes’ PROCESS macro (version 5.0, model 4). No missing data or multivariate outliers were detected based on boxplots, Cook’s distance, the Mahalanobis distance, or leverage statistics. Pearson’s *r* was used to estimate correlations between quantitative variables [110]. All analyses were performed using a 95% confidence interval and 5000 bootstrap samples [111]. A priori power analysis was conducted in G*Power version 3.1.9.4 (Heinrich-Heine-Universität, Düsseldorf, Germany) using a bivariate normal correlation model [112] to estimate the required sample size. We specified a small effect size of 0.21, identified by Richard et al.’s [113] as the average effect size across published studies, along with an alpha level of 0.05, and a desired power of 0.90. The analysis indicated that a minimum sample of 187 participants was required.

## 3. Results

### 3.1. Basic Descriptive Characteristics

Before the main statistical analyses, descriptive statistics and the Shapiro–Wilk test were performed to assess the degree to which the assumption of normality was met for the distributions of the analyzed variables (Table 2).

The results indicated that only the distribution of loneliness did not differ significantly from a normal distribution (W(207) = 0.99, *p* > 0.05). Although the Shapiro–Wilk tests showed statistically significant deviations from normality for the remaining variables, their skewness and kurtosis values fell within the ±1 range. These indices suggest that the deviations are not severe enough to invalidate parametric analyses [114]. Moreover, to ensure the robustness of the results against potential violations of normality assumptions, bootstrapping with 5000 samples was applied in all subsequent analyses.

### 3.2. Correlation Analysis

To test the hypotheses assuming the co-occurrence of the psychological variables (H1–H3), a Pearson’s *r* correlation analysis was conducted (Table 3).

The results revealed statistically significant correlations (*p* < 0.01) between all pairs of variables. The strongest negative association was observed between loneliness and life satisfaction (*r* = −0.50; *p* < 0.001; 95% *CI* [−0.61, −0.38]). In addition, life satisfaction was negatively correlated with indirect self-destructive behavior (*r* = −0.31; *p* < 0.001; 95% *CI* [−0.44, −0.18]). A weak but significant positive correlation was also found between loneliness and indirect self-destructive behavior (*r* = 0.20; *p* < 0.01; 95% *CI* [0.06, 0.34]). In other words, higher levels of loneliness and indirect self-destructive behavior were associated with lower levels of life satisfaction. Moreover, it can be acknowledged that increased indirect self-destructive behavior is related to elevated levels of loneliness. These findings provide preliminary support for further testing of the mediating role of loneliness in the relationship between indirect self-destructive behavior and life satisfaction.

### 3.3. Mediating Role of Loneliness Between Indirect Self-Destructive Behavior and Life Satisfaction

**Figure 1:**
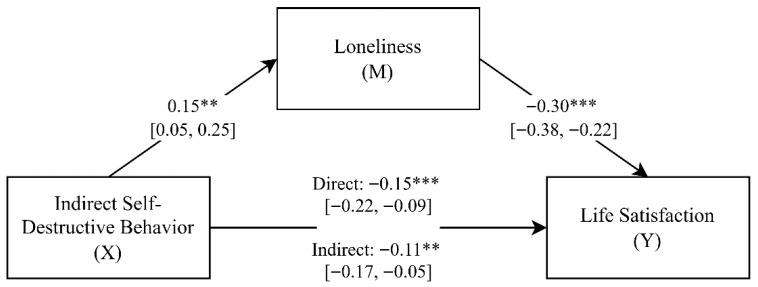
Mediation model illustrating the indirect effect of indirect self-destructive behavior on life satisfaction through loneliness (standardized regression coefficients) (*N* = 207). ** *p* < 0.01; *** *p* < 0.001.

To estimate the mediating role of loneliness in the relationship between indirect self-destructive behavior and life satisfaction, a mediation analysis was carried out, with the results presented in Figure 1.

The estimated model was well-fitted to the data (*F*(2, 204) = 43.60; *p* < 0.001) and explained approximately 30% of the variance in life satisfaction (*adjR*^2^ = 0.30). The obtained standardized regression coefficients indicated that indirect self-destructive behavior was positively associated with loneliness (β = 0.15; *p* < 0.01; 95% *CI* [0.05, 0.25]), while loneliness had a negative impact on life satisfaction (β = −0.30; *p* < 0.001; 95% *CI* [−0.38, −0.22]). Consequently, increases in both indirect self-destructive behavior and loneliness were linked to lower life satisfaction.

Furthermore, the direct effect of indirect self-destructive behavior on life satisfaction (β = −0.15; *p* < 0.001; 95% *CI* [−0.22, −0.09]) was attenuated when accounting for the mediating role of loneliness (β = −0.11; *p* < 0.01; 95% *CI* [−0.17, −0.05]). The significant indirect effect and the absence of zero within the bootstrap 95% confidence interval (*Indirect* = −0.05; 95% *CI* [−0.08, −0.01]) provide evidence of a statistically significant partial mediation [74]. In other words, indirect self-destructive behavior increases feelings of loneliness, which decreases life satisfaction.

In the next step, a mediation analysis was conducted to examine the mediating role of loneliness in the relationship between indirect self-destructive behavior and life satisfaction, while controlling for covariates. When controlling for age (β = −0.06; 95% *CI* [−0.14, 0.02]), place of residence (β = 0.28; 95% *CI* [−0.23, 0.79]), and the type of family dysfunction (β = 0.01; 95% *CI* [−0.22, 0.23]), the pattern of mediation remained unchanged (*F*(5, 201) = 18.22; *p* < 0.001; *adjR*^2^ = 0.31; total effect: β = −0.17; *p* < 0.001; 95% *CI* [−0.23, −0.11]; direct effect: β = −0.12; *p* < 0.01; 95% *CI* [−0.18, −0.06]), and none of the covariates significantly predicted life satisfaction (*p* > 0.05). This indicates that the indirect effect of indirect self-destructive behavior via loneliness is robust to these demographic factors, confirming that loneliness partially mediates the relationship (*Indirect* = −0.05; 95% *CI* [−0.08, −0.02]) independently of age, place of residence, and the type of family dysfunction.

### 3.4. Mediating Role of Indirect Self-Destructive Behavior Between Loneliness and Life Satisfaction

**Figure 2:**
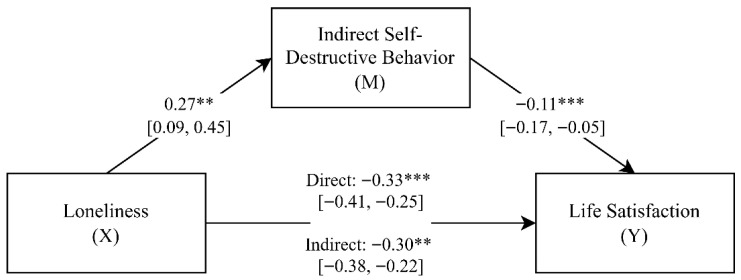
Conceptual mediation model illustrating the indirect effect of loneliness on life satisfaction through indirect self-destructive behavior (standardized regression coefficients) (*N* = 207). ** *p* < 0.01; *** *p* < 0.001.

In line with the adoption of a circular perspective on the relationship between indirect self-destructive behavior and loneliness, we also tested an alternative mediation model, where loneliness was the independent variable and indirect self-destructive behavior as a mediator. To estimate the mediating role of indirect self-destructive behavior in the relationship between loneliness and life satisfaction, a mediation analysis was carried out, with the results presented in Figure 2.

The estimated model was well-fitted to the data (*F*(2, 204) = 43.60; *p* < 0.001) and explained approximately 29% of the variance in life satisfaction (*adjR*^2^ = 0.29). The obtained standardized regression coefficients indicated that loneliness was positively associated with indirect self-destructive behavior (β = 0.27; *p* < 0.01; 95% *CI* [0.09, 0.45]), while indirect self-destructive behavior was negatively associated with life satisfaction (β = −0.11; *p* < 0.001; 95% *CI* [−0.17, −0.05]). These results suggest that higher levels of loneliness are linked with self-destructive tendencies, which in turn are associated with decreased life satisfaction.

Additionally, the direct effect of loneliness on life satisfaction (β = −0.33; *p* < 0.001; 95% *CI* [−0.41, −0.25]) was partially reduced after including the mediator (β = −0.30; *p* < 0.001; 95% *CI* [−0.38, −0.22]). The presence of a statistically significant indirect effect (*Indirect* = −0.03; 95% *CI* [−0.06, −0.01]) and a bootstrap confidence interval that did not include zero confirms the presence of partial mediation [74]. In other words, loneliness diminishes life satisfaction in part through its adverse impact on self-destructive tendencies.

When the same mediation model was estimated with age (β = −0.06; 95% *CI* [−0.14, 0.02), place of residence (β = 0.28; 95% *CI* [−0.23, 0.79]), and type of family dysfunction (β = 0.01; 95% *CI* [−0.22, 0.23]) entered as covariates, the overall mediation pattern remained consistent with the initial results. None of the covariates significantly predicted life satisfaction (*p* > 0.05), and their inclusion did not substantially alter the relationships among the main study variables. The indirect effect of loneliness through indirect self-destructive behavior remained statistically significant (*Indirect* = −0.03; 95% *CI* [−0.07, −0.01]), indicating that the mediating mechanism is robust to these demographic factors. The total (β = −0.32; *p* < 0.001; 95% *CI* [−0.40, −0.24]) and direct (β = −0.29; *p* < 0.001; 95% *CI* [−0.36, −0.21]) effects of loneliness on life satisfaction were only slightly reduced, further supporting the presence of partial mediation independent of the included covariates.

## 4. Discussion

This study addressed the limited empirical attention to the interplay between indirect self-destructive behavior, loneliness, and life satisfaction among women experiencing different forms of family dysfunction. Specifically, we examined the association between indirect self-destructive behavior and life satisfaction, assessed whether loneliness mediates this relationship, and tested an alternative mediation model in which loneliness serves as the predictor and indirect self-destructive behavior as the mediator.

Consistent with hypothesis H1, our findings confirm a negative association between indirect self-destructive behavior and women’s cognitive evaluations of their overall quality of life. Given that indirect self-destructiveness represents a more subtle and disguised form of self-harm [115], encompassing behaviors that may adversely affect one’s life, it is not surprising that risky behaviors, addictions, and various forms of self-neglect increase the likelihood of future negative consequences. As noted by several researchers [25,26,116], such behaviors are believed to carry long-term detrimental effects, including a reduced quality of life and even a shortened lifespan.

Our study also demonstrated a positive association between indirect self-destructive behavior and loneliness, thereby supporting hypothesis H2. This relationship suggests that, for some women, indirect self-harm behaviors may function as a means of expressing distress or signaling a need for support [117]. Prior research by Troya et al. [118] indicates that older adults experience heightened loneliness following episodes of self-harm, while those close to them may respond with feelings of shame. A similar lack of understanding and social support may arise in the context of women’s indirect self-destructiveness, particularly when such behaviors are accompanied by social withdrawal. Moreover, indirect self-destructive actions can evoke feelings of shame, which, as noted by several authors, may contribute to “thwarted belonging and perceived burdensomeness” [119] (p. 4).

Hypothesis H3 was also supported, indicating that loneliness is negatively associated with life satisfaction. This finding aligns with previous research showing that individuals who experience loneliness tend to report lower levels of life satisfaction [120,121,122,123,124]. Within the framework of social determinism, life satisfaction, a key component of subjective well-being, is strongly tied to the fundamental human need for meaningful social connections [125]. Loneliness, understood as the emotional response to a perceived gap between desired and actual social relationships [72], is further linked to hopelessness and depressive symptoms [126,127]. Given these associations, it is unsurprising that loneliness emerges as a significant predictor and vulnerability factor for diminished life satisfaction.

The most important outcome of our research is the confirmation of hypothesis H4. Although the mediation results must be interpreted cautiously due to the cross-sectional design, the findings suggest that among women who experienced dysfunction in their family of origin, indirect self-destructive behaviors are associated with reduced psychological well-being partly through their connection with distress arising from perceived deficits in social relationships [120]. This pattern is understandable given that growing up in families marked by conflict or emotional and physical abuse may reinforce both self-destructive tendencies [128] and feelings of loneliness, each of which can adversely affect well-being [129]. While consistent with theoretical models positioning loneliness as a potential mechanism linking self-destructiveness to diminished well-being, the current design does not permit conclusions about temporal or causal pathways. Thus, the results should be viewed as associations rather than evidence that indirect self-destructiveness leads to loneliness or lower life satisfaction. Longitudinal or experimental studies are needed to determine whether these relations reflect causal processes or alternative reciprocal dynamics.

It is important to note that although the observed associations were statistically significant, they were modest in magnitude. Zero-order correlations accounted for approximately 4–10% of shared variance, consistent with typical effect sizes in psychosocial research [130]. Small effects may nevertheless be meaningful when considered cumulatively or within populations exposed to chronic stress. In line with recommendations for interpreting mediation models [131], we evaluated not only the variance explained but also the size of the indirect pathways, acknowledging that mediated effects are commonly smaller than their bivariate counterparts. The present indirect effect therefore reflects a modest but theoretically coherent pathway linking indirect self-destructive behavior, loneliness, and life satisfaction. From a clinical perspective, however, the size of the indirect effects suggests that loneliness explains only a small proportion of the association between indirect self-destructiveness and life satisfaction. Although small indirect effects are common in multifactorial psychological constructs, these findings should not be overinterpreted. Stronger conclusions about underlying mechanisms will require longitudinal and clinically informed research.

Finally, alternative explanations must be considered. Childhood trauma predicts loneliness, self-destructive tendencies, and reduced well-being independently [132], suggesting a shared underlying vulnerability. Personality traits, particularly neuroticism, are also associated with loneliness, maladaptive coping, and low life satisfaction [133,134]. Selection effects may further contribute, as individuals who engage in self-destructive behaviors often isolate themselves or enter environments that reinforce social withdrawal [135]. Given these possibilities, the present mediation results represent an associational pattern rather than a definitive mechanism.

Although briefly, we would also like to note that our study confirmed hypothesis H5, indicating that loneliness is associated with life satisfaction via indirect self-destructive behaviors. This finding is consistent with previous research showing that feelings of loneliness are among the risk factors for dysfunctional coping [136], which, in turn, are linked to the overall well-being of individuals who experience them [33]. It may therefore be assumed that working with women, and more broadly, with individuals, who grew up in dysfunctional families should involve strengthening their social support networks to reduce feelings of loneliness, as well as identifying subtle, often overlooked behaviors that hinder personal growth. These forms of intervention may contribute to improvements in their overall psychological and social functioning.

## 5. Limitations and Future Directions

Several limitations warrant consideration. First, consistent with the adopted research model, the assessment was conducted only among women. Future studies should expand the sample to include men, allowing gender-based comparisons and improving representativeness. Second, the sociodemographic characteristics assessed were limited to basic variables such as age and place of residence, preventing more detailed analyses of factors like education, marital status, or parental status, which may be important given their associations with social support. Importantly, the study did not account for participants’ current living situation (living alone vs. with others), a variable directly relevant to loneliness [75]. Furthermore, factors such as therapy or counseling history and the time elapsed since leaving the family of origin were not measured, despite their potential to mitigate the impact of early adversity. Future research should therefore incorporate these detailed sociodemographic, psychosocial, and clinical characteristics, including both perceived and received social support, to more fully explain the mechanisms underlying indirect self-destructive behavior.

Additionally, although the current study reported correlations for social and emotional loneliness subscales, the mediation analyses relied on the global loneliness score. Future research should examine whether these dimensions mediate the relationship between indirect self-destructive behavior and life satisfaction differentially, as this could reveal distinct pathways of influence.

Another limitation concerns the measurement of family dysfunction. Because it was assessed using self-classification questions without additional verification, the responses reflect subjective perceptions rather than objectively validated indicators. Future studies would benefit from using standardized instruments (e.g., the Family Assessment Device, the Dysfunctional Attitude Scale). Recruitment strategy also poses limitations: participants were recruited through Facebook groups, online forums, and support communities for individuals raised in dysfunctional families. This may introduce selection bias, as women who seek online support may differ systematically from those who do not, potentially experiencing higher distress or greater help-seeking tendencies. As a result, the sample may not fully represent the broader population of women from dysfunctional families. Future studies should use more diverse recruitment channels, including clinical settings, community organizations, and population-based samples. Given that age was included solely as a control variable (covariate) and was not examined as a moderator, future research may consider modeling age as a moderating variable to examine whether differences attributable to developmental stage are present.

A major methodological limitation of the present study is the use of cross-sectional data to test mediation models, which inherently assume temporal ordering. Although we tested an alternative model to illustrate the potential bidirectionality of the associations, cross-sectional mediation cannot establish whether the predictor precedes the mediator or whether the mediator precedes the outcome. Thus, causal interpretations are not warranted, and reverse causation remains possible. Moreover, reliance on self-report questionnaires administered at a single time point introduces the risk of common method variance, which may inflate observed associations. To overcome these limitations, future research should employ longitudinal or experimental designs, repeated measurement of key variables, and multi-method data sources such as clinical interviews or behavioral assessments.

Finally, although the indirect effects identified in this study were statistically significant, their magnitude was very small. This indicates that a one-unit increase in indirect self-destructive behavior corresponds to only minimal changes in life satisfaction through loneliness. From a clinical perspective, such effects should be interpreted with caution. At the same time, small indirect effects are common in multifactorial psychological models and may nevertheless be meaningful when accumulated across time or within high-risk populations [130. Still, the modest size of these pathways suggests that loneliness explains only a limited portion of the association between indirect self-destructiveness and life satisfaction. Thus, while the mediation model provides theoretically coherent insight into a possible mechanism, stronger conclusions will require longitudinal and clinically grounded research.

## 6. Conclusions

Despite these limitations, the present study addresses an important gap in the literature by examining indirect self-destructive behaviors, loneliness, and their links to life satisfaction among women with dysfunctional family backgrounds. The findings provide valuable insights for interdisciplinary teams working with adults and suggest concrete practical implications, including the need to screen for loneliness in family therapy settings and to strengthen social connectedness in interventions targeting indirect self-destructive patterns, such as self-neglect, intentional suffering, helplessness, or risky behaviors. Attending to these manifestations is essential, as unprocessed indirect self-destructiveness may escalate into direct self-harm, and because dysfunction within families is often intergenerationally transmitted [6].

## Figures and Tables

**Table 1:** Description of the type of dysfunction present in the women’s families of origin (*N* = 207).

Type of Family Dysfunction	Prevalence (*N*) Percent
psychological violence (humiliation, instilling fear, limiting social contacts, imposing one’s own views)	(59) 28.5%
alcoholism	(55) 26.6%
nervous and/or depressive home atmosphere (tense, full of distrust, dominated by sadness, resignation)	(32) 15.5%
increased, often unresolved family conflicts	(18) 8.7%
parents’ marital conflict	(12) 5.8%
indifference in relations between members, lack of emotional support or satisfaction of emotional needs	(8) 3.9%
breakdown of the family structure	(8) 3.9%
physical violence	(7) 3.4%
lack of care, control, and responsibility of a parent over a child	(3) 1.4%
sexual violence	(3) 1.4%
drug addiction	(2) 0.9%

**Table 2:** Descriptive statistics and results of the Shapiro–Wilk normality test (*N* = 207).

Variable	*M*	*SD*	Skewness	Kurtosis	W
Loneliness	60.88	13.43	0.12	−0.22	0.99
Social dimension	16.04	5.00	−0.25	−0.50	0.98 **
Emotional dimension	18.86	5.98	−0.10	−0.84	0.98 **
Life Satisfaction	15.78	6.59	0.50	−0.24	0.97 ***
Indirect Self-Destructive Behavior	34.90	10.09	−0.14	−0.63	0.99 *

W—Shapiro–Wilk statistic; * *p* < 0.05; ** *p* < 0.01; *** *p* < 0.001.

**Table 3:** Pearson’s r coefficient values between the analyzed psychological variables (*N* = 207).

	(1)	(2)	(3)	(4)
Loneliness (1)	-			
Social dimension (2)	0.90 *** [0.86, 0.93]	-		
Emotional dimension (3)	0.93 *** [0.91, 0.95]	0.69 *** [0.58, 0.77]	-	
Life Satisfaction (4)	−0.50 *** [−0.61, −0.38]	−0.47 *** [−0.58, −0.35]	−0.45 *** [−0.56, −0.32]	-
Indirect Self-Destructive Behavior (5)	0.20 ** [0.06, 0.34]	0.18 ** [0.04, 0.32]	0.18 ** [0.05, 0.32]	−0.31 *** [−0.44, −0.18]

** *p* < 0.01; *** *p* < 0.001.

## Data Availability

The dataset analyzed during the present study is available in the OSF repository and can be accessed at https://osf.io/e5fst/?view_only=e9e63ce9a2be4c0085eaee853d509ad6, accessed on 14 December 2025.

## References

[1] Mushkevych M. (2020). Psychological Characteristics of Emotional Functioning of Families with Problem Children. Ann. Univ. Mariae Curie-Skłodowska.

[2] Zhou Q., Lin C., Guo X. (2025). Family Dysfunction, Parenting Stress, and Child Mental Health: Associations with Bullying Involvement and the Moderating Role of Neighborhood Support. Front. Psychol..

[3] Ariel S. (1987). An Information Processing Theory of Family Dysfunction. Psychotherapy.

[4] Malagón-Amor Á., Martín-López L.M., Córcoles D., González A., Bellsolà M., Teo A.R., Bulbena A., Pérez V., Bergé D. (2020). Family Features of Social Withdrawal Syndrome (Hikikomori). Front. Psychiatry.

[5] Huang X.-C., Zhang Y.-N., Wu X.-Y., Jiang Y., Cai H., Deng Y.-Q., Luo Y., Zhao L.-P., Liu Q.-L., Luo S.-Y. (2023). A Cross-Sectional Study: Family Communication, Anxiety, and Depression in Adolescents: The Mediating Role of Family Violence and Problematic Internet Use. BMC Public Health.

[6] Zagefka H., Jones J., Caglar A., Girish R., Matos C. (2021). Family Roles, Family Dysfunction, and Depressive Symptoms. Fam. J..

[7] Kawamura K., Frost R.O., Harmatz M.G. (2002). The Relationship of Perceived Parenting Styles to Perfectionism. Pers. Individ. Dif..

[8] Morelli N., Hong K., Elzie X., Garcia J. (2022). Bidirectional Associations between Family Conflict and Child Behavior Problems in Families at Risk for Maltreatment. Child Abus. Negl..

[9] Kenyhercz V., Frikker G., Kaló Z., Demetrovics Z., Kun B. (2022). Dysfunctional Family Mechanisms, Internalized Parental Values, and Work Addiction: A Qualitative Study. Sustainability.

[10] Reymundo-Sánchez B.L., Matías-Pérez D., Mendoza-García C.A., García-Montalvo I.A. (2025). Prevalence of Suicidal Ideation, Depression, and Family Dysfunction in First-Year Students of the Bachelor’s Degree in Medical Surgery at the Universidad Regional del Sureste. Front. Educ..

[11] Wang Y., Tian L., Guo L., Huebner E.S. (2020). Family Dysfunction and Adolescents’ Anxiety and Depression: A Multiple Mediation Model. J. Appl. Dev. Psychol..

[12] Calaresi D., Verrastro V., Giordano F., Saladino V. (2025). The Long-Term Effects of Emotional Neglect on Family Functioning and Anxiety Symptoms in Adolescents. J. Fam. Viol..

[13] Allen J., Moore J. (2017). Troubling the Functional/Dysfunctional Family Binary through the Articulation of Functional Family Estrangement. West. J. Commun..

[14] Ryff C.D., Keyes C.L.M. (1995). The Structure of Psychological Well-Being Revisited. J. Pers. Soc. Psychol..

[15] Ryff C.D., Singer B. (1998). The Contours of Positive Human Health. Psychol. Inq..

[16] Tamir M., Gross J.J., Sheldon K.M., Kashdan T.B., Steger M.F. (2011). Beyond Pleasure and Pain? Emotion Regulation and Positive Psychology. Designing Positive Psychology: Taking Stock and Moving Forward.

[17] Wong P.T.P., Tomer A. (2011). Beyond Terror and Denial: The Positive Psychology of Death Acceptance. Death Stud..

[18] Botha F., Booysen F. (2014). Family Functioning and Life Satisfaction and Happiness in South African Households. Soc. Indic. Res..

[19] Izzo F., Baiocco R., Pistella J. (2022). Children’s and Adolescents’ Happiness and Family Functioning: A Systematic Literature Review. Int. J. Environ. Res. Public Health.

[20] Tsirigotis K. (2016). Indirect Self-Destructiveness and Emotional Intelligence. Psychiatr. Q..

[21] Tsirigotis K. (2019). Gender Differentiation of Indirect Self-Destructiveness in Drug Addicted Individuals (Indirect Self-Destructiveness in Addicted Women and Men). Psychiatr. Q..

[22] Tsirigotis K., Łuczak J. (2018). Indirect Self-Destructiveness in Women Who Experience Domestic Violence. Psychiatr. Q..

[23] Tsirigotis K., Gruszczyński W., Kruszyna M., Tsirigotis-Wołoszczak M. (2009). Autodestruktywność Pośrednia u Osób Uzależnionych od Narkotyków. Alcohol Drug Addict..

[24] Farberow N.L. (1980). The Many Faces of Suicide: Indirect Self-Destructive Behavior.

[25] Kelley K., Byrne D., Przybyla D.P.J., Eberly C., Eberly B., Greendlinger V., Wan C.K., Gorsky J. (1985). Chronic Self-Destructiveness: Conceptualization, Measurement, and Initial Validation of the Construct. Motiv. Emot..

[26] Suchańska A. (1998). Przejawy i Uwarunkowania Psychologiczne Pośredniej Autodestruktywności.

[27] Baumeister R.F., Scher S.J. (1988). Self-Defeating Behavior Patterns among Normal Individuals: Review and Analysis of Common Self-Destructive Tendencies. Psychol. Bull..

[28] Okasha A., Pichot P., Berner P., Wolf R., Thau K. (1985). The Self-Destructive Behaviour of Everyday Life. Clinical Psychopathology Nomenclature and Classification.

[29] Conwell Y., Pearson J., DeRenzo E.G. (1996). Indirect Self-Destructive Behavior among Elderly Patients in Nursing Homes: A Research Agenda. Am. J. Geriatr. Psychiatry.

[30] Pompili M., Mancinelli I., Girardi P., Kotzalidis G., Lazanio S., Tatarelli R. (2004). Risk-Taking Behaviour, Subintentioned Death and Suicide. J. Dev. Behav. Pediatr..

[31] Hosseini C., Walsh J., Brown L.M., Kumar U. (2017). Indirect Self-Destructive Behaviour across the Lifespan. Handbook of Suicidal Behaviour.

[32] Nelson F.L., Farberow N.L. (1980). Indirect Self-Destructive Behavior in the Elderly Nursing Home Patient. J. Gerontol..

[33] Ekramzadeh S., Javadpour A., Draper B., Mani A., Withall A., Sahraian A. (2012). Prevalence and Correlates of Suicidal Thought and Self-Destructive Behavior among an Elderly Hospital Population in Iran. Int. Psychogeriatr..

[34] Głowaczewska A., Reszke R., Szepietowski J.C., Matusiak Ł. (2021). Indirect Self-Destructiveness in Hidradenitis Suppurativa Patients. J. Clin. Med..

[35] Tsirigotis K., Gruszczyński W., Tsirigotis-Wołoszczak M. (2010). Indirect (Chronic) Self-Destructiveness and Modes of Suicide Attempts. Arch. Med. Sci..

[36] Sharp M., Schill T. (1995). Chronic Self-Destructiveness and Self-Defeating Personality: Similarities and Differences. J. Pers. Assess..

[37] Sławińska A., Orzechowska A., Florkowski A. (2018). Assessment of the Occurrence of Indirect Self-Destructiveness in People Diagnosed with Depressive and Anxiety Disorders. Psychiatr. Psychol. Klin..

[38] Lee D.E. (1985). Alternative Self-Destruction. Percept. Mot. Ski..

[39] Firestone R.W., Firestone L. (1998). Voices in Suicide: The Relationship between Self-Destructive Thought Processes, Maladaptive Behavior, and Self-Destructive Manifestations. Death Stud..

[40] Szanto K., Shear M.K., Houck P.R., Reynolds C.F., Frank E., Caroff K., Silowash R. (2006). Indirect Self-Destructive Behavior and Overt Suicidality in Patients with Complicated Grief. J. Clin. Psychiatry.

[41] Gerber K.E., Nehemkis A.M., Farberow N.L., Williams J. (1981). Indirect Self-Destructive Behavior in Chronic Hemodialysis Patients. Suicide Life Threat. Behav..

[42] Draper B., Brodaty H., Low L.F., Richards V., Paton H., Lie D. (2002). Self-Destructive Behaviors in Nursing Home Residents. J. Am. Geriatr. Soc..

[43] McIntosh J.L., Hubbard R.W. (1988). Indirect Self-Destructive Behavior among the Elderly: A Review with Case Examples. J. Gerontol. Soc. Work.

[44] Farberow N.L., Nehemkis A.M. (1979). Indirect Self-Destructive Behavior in Patients with Buerger’s Disease. J. Pers. Assess..

[45] Grandclerc S., De Labrouhe D., Spodenkiewicz M., Lachal J., Moro M.-R. (2016). Relations between Nonsuicidal Self-Injury and Suicidal Behavior in Adolescence: A Systematic Review. PLoS ONE.

[46] Gagnon J., Daelman S. (2011). An Empirical Study of the Psychodynamics of Borderline Impulsivity: A Preliminary Report. Psychoanal. Psychol..

[47] Villagrán A., Lund C., Duncan R., Lossius M.I. (2022). The Effect of Attachment Style on Long-Term Outcomes in Psychogenic Nonepileptic Seizures: Results from a Prospective Study. Epilepsy Behav..

[48] Cerutti R., Presaghi F., Manca M., Gratz K.L. (2012). Deliberate Self-Harm Behavior among Italian Young Adults: Correlations with Clinical and Nonclinical Dimensions of Personality. Am. J. Orthopsychiatry.

[49] Cruz D., Narciso I., Pereira C., Sampaio D. (2015). Self-Destructive Symptomatic Frames in Clinical Adolescents: Is the Same Different?. J. Res. Adolesc..

[50] Tsirigotis K., Gruszczyński W., Lewik-Tsirigotis M. (2013). Manifestations of Indirect Self-Destructiveness and Methods of Suicide Attempts. Psychiatr. Q..

[51] Sinclair J.M., Hawton K., Gray A. (2010). Six-Year Follow-Up of a Clinical Sample of Self-Harm Patients. J. Affect. Disord..

[52] Mitchell E., Rosario-Williams B., Yeshchenko I., Miranda R. (2023). Cognitive Emotion Regulation Strategies among Emerging Adults with Different Self-Harm Histories. J. Affect. Disord. Rep..

[53] Kong F., You X. (2013). Loneliness and Self-Esteem as Mediators between Social Support and Life Satisfaction in Late Adolescence. Soc. Indic. Res..

[54] Milovanska-Farrington S., Farrington S. (2022). Happiness, Domains of Life Satisfaction, Perceptions, and Valuation Differences across Genders. Acta Psychol..

[55] Popov I.V. (2002). A Concept of Self-Destructive Behavior in Adolescents. Int. J. Ment. Health.

[56] Gunnarsson N.V. (2025). “Going Beyond Darkness”—Lingering Images and Ideation of Self-Destruction. J. Psychiatr. Ment. Health Nurs..

[57] Rosales K., Wendel Rice E., Brown L.M., Pompili M. (2022). Indirect Self-Destructive Behaviors. Suicide Risk Assessment and Prevention.

[58] Qian G. (2019). Associations of Suicide and Subjective Well-Being. OMEGA-J. Death Dying.

[59] Kleszczewska-Albińska A. (2022). Self-Destructive Behaviors, Self-Esteem, Anxiety, and Social Desirability in People with Personality and Mood Disorders. InPACT.

[60] Boudewyn A.C., Liem J.H. (1995). Psychological, Interpersonal, and Behavioral Correlates of Chronic Self-Destructiveness: An Exploratory Study. Psychol. Rep..

[61] Huebner E.S., Dew T. (1996). The Interrelationships of Positive Affect, Negative Affect, and Life Satisfaction in an Adolescent Sample. Soc. Indic. Res..

[62] Garcia D., Archer T. (2012). Adolescent Life Satisfaction and Well-Being. J. Altern. Med. Res..

[63] Rönkä A.R., Taanila A., Koiranen M., Sunnari V., Rautio A. (2013). Associations of Deliberate Self-Harm with Loneliness, Self-Rated Health and Life Satisfaction in Adolescence: Northern Finland Birth Cohort 1986 Study. Int. J. Circumpolar Health.

[64] Le N., Belay Y.B., Le L.K., Pirkis J., Mihalopoulos C. (2023). Health-Related Quality of Life in Children, Adolescents and Young Adults with Self-Harm or Suicidality: A Systematic Review. Aust. N. Z. J. Psychiatry.

[65] Rodgers B. (1991). Models of Stress, Vulnerability and Affective Disorder. J. Affect. Disord..

[66] Rudd M.D., Brickell M. (2009). Depression and Suicide: A Diathesis–Stress Model for Understanding and Treatment. Living and Surviving in Harm’s Way.

[67] Thurston H., Bell J.F., Induni M. (2018). Community-Level Adverse Experiences and Emotional Regulation in Children and Adolescents. J. Pediatr. Nurs..

[68] Karatekin C., Ahluwalia R. (2020). Effects of Adverse Childhood Experiences, Stress, and Social Support on the Health of College Students. J. Interpers. Violence.

[69] Cruz D., Narciso I., Muñoz M., Pereira C.R., Sampaio D. (2013). Adolescents and Self-Destructive Behaviours: An Exploratory Analysis of Family and Individual Correlates. Behav. Psychol./Psicol. Conduct..

[70] Li X., Zhou Y., Liu L. (2024). Relationship between Loneliness, Hopelessness, Coping Style, and Mobile Phone Addiction among Non-Suicidal Self-Injury Adolescents. Psychol. Res. Behav. Manag..

[71] Calati R., Ferrari C., Brittner M., Oasi O., Olié E., Carvalho A.F., Courtet P. (2018). Suicidal Thoughts and Behaviors and Social Isolation: A Narrative Review of the Literature. J. Affect. Disord..

[72] Perlman D., Peplau L.A. (1981). Toward a Social Psychology of Loneliness. Pers. Relatsh..

[73] Geulayov G., Mansfield K., Jindra C., Hawton K., Fazel M. (2024). Loneliness and Self-Harm in Adolescents during the First National COVID-19 Lockdown: Results from a Survey of 10,000 Secondary School Pupils in England. Curr. Psychol..

[74] John A., Lee S.C., Solomon S., Crepaz-Keay D., McDaid S., Morton A., Davidson G., Van Bortel T., Kousoulis A.A. (2021). Loneliness, Coping, Suicidal Thoughts and Self-Harm during the COVID-19 Pandemic: A Repeat Cross-Sectional UK Population Survey. BMJ Open.

[75] Shaw R.J., Cullen B., Graham N., Lyall D.M., Mackay D., Okolie C., Pearsall R., Ward J., John A., Smith D.J. (2021). Living Alone, Loneliness and Lack of Emotional Support as Predictors of Suicide and Self-Harm: A Nine-Year Follow-Up of the UK Biobank Cohort. J. Affect. Disord..

[76] Titelman D. (2006). Primo Levi’s Loneliness: Psychoanalytic Perspectives on Suicide-Nearness. Psychoanal. Q..

[77] Yavuzer Y., Albayrak G., Kılıçarslan S. (2018). Relationships among Aggression, Self-Theory, Loneliness, and Depression in Emerging Adults. Psychol. Rep..

[78] Van Orden K.A., Witte T.K., Cukrowicz K.C., Braithwaite S.R., Selby E.A., Joiner T.E. (2010). The Interpersonal Theory of Suicide. Psychol. Rev..

[79] Radziwiłłowicz W., Grzegorzewska I., Cierpiałkowska L., Borkowska A.R. (2020). Autoagresja—Samobójstwa i Samookaleczenia. Psychologia Kliniczna Dzieci i Młodzieży.

[80] Kaufman E., Alterman A.I. (1985). Family Adaptation to Substance Abuse. Substance Abuse and Psychopathology.

[81] Jia Y., Yue Y., Sheng Y. (2025). The Mediating Role of Aging Attitudes between Social Isolation and Self-Neglect: A Cross-Sectional Study of Older Adults Living Alone in Rural China. BMC Nurs..

[82] Jones W.H., Freemon J.E., Goswick R.A. (1981). The Persistence of Loneliness: Self and Other Determinants. J. Pers..

[83] Besser A., Flett G.L., Davis R.A. (2003). Self-Criticism, Dependency, Silencing the Self, and Loneliness: A Test of a Mediational Model. Pers. Individ. Dif..

[84] Ingram I., Kelly P.J., Deane F.P., Baker A.L., Goh M.C.W., Raftery D.K., Dingle G.A. (2020). Loneliness among People with Substance Use Problems: A Narrative Systematic Review. Drug Alcohol Rev..

[85] Geulayov G., Metcalfe C., Heron J., Kidger J., Gunnell D. (2014). Parental Suicide Attempt and Offspring Self-Harm and Suicidal Thoughts: Results from the Avon Longitudinal Study of Parents and Children (ALSPAC) Birth Cohort. J. Am. Acad. Child Adolesc. Psychiatry.

[86] Cawley R., Pontin E.E., Touhey J., Sheehy K., Taylor P.J. (2019). What is the relationship between rejection and self-harm or sui-cidality in adulthood?. J. Affect. Disord..

[87] Paul E., Kwong A., Moran P., Pawlby S., Howard L.M., Pearson R.M. (2021). Maternal Thoughts of Self-Harm and Their Association with Future Offspring Mental Health Problems. J. Affect. Disord..

[88] Troya M.I., Dikomitis L., Babatunde O.O., Bartlam B., Chew-Graham C.A. (2019). Understanding Self-Harm in Older Adults: A Qualitative Study. EClinicalMedicine.

[89] Mikulincer M., Shaver P.R. (2010). Attachment in Adulthood: Structure, Dynamics, and Change.

[90] Vanhalst J., Luyckx K., Raes F., Goossens L. (2012). Loneliness and Depressive Symptoms: The Mediating and Moderating Role of Uncontrollable Ruminative Thoughts. J. Psychol..

[91] Lacey R.E., Bartley M., Pikhart H., Stafford M., Cable N. (2014). Parental Separation and Adult Psychological Distress: An Investigation of Material and Relational Mechanisms. BMC Public Health.

[92] Shevlin M., McElroy E., Murphy J. (2015). Loneliness Mediates the Relationship between Childhood Trauma and Adult Psychopathology: Evidence from the Adult Psychiatric Morbidity Survey. Soc. Psychiatry Psychiatr. Epidemiol..

[93] Georgeson A.R., Alvarez-Bartolo D., MacKinnon D.P. (2025). A Sensitivity Analysis for Temporal Bias in Cross-Sectional Mediation. Psychol. Methods.

[94] Wedajo L.F., Hajure M., Abdu Z., Tesfaye G.M., Workneh Y.A., Gezimu W., Hussen M.A., Gemeda A.D., Teferi S.M., Alemu S.S. (2024). Magnitude of Self-Harm and Associated Factors among Postnatal Mothers Attending Immunization Clinics at Public Health Facilities in Boneya Boshe Woreda, Western Ethiopia, 2023: Institution-Based Cross-Sectional Study Design. Front. Public Health.

[95] Mellor D., Stokes M., Firth L., Hayashi Y., Cummins R. (2008). Need for Belonging, Relationship Satisfaction, Loneliness, and Life Satisfaction. Pers. Individ. Dif..

[96] Gan S.W., Ong L.S., Lee C.H., Lin Y.S. (2020). Perceived Social Support and Life Satisfaction of Malaysian Chinese Young Adults: The Mediating Effect of Loneliness. J. Genet. Psychol..

[97] Kekkonen V., Tolmunen T., Kraav S.-L., Hintikka J., Kivimäki P., Kaarre O., Laukkanen E. (2020). Adolescents’ Peer Contacts Promote Life Satisfaction in Young Adulthood—A Connection Mediated by the Subjective Experience of Not Being Lonely. Pers. Individ. Dif..

[98] Stepanikova I., Nie N.H., He X. (2010). Time on the Internet at Home, Loneliness, and Life Satisfaction: Evidence from Panel Time-Diary Data. Comput. Hum. Behav..

[99] Kaya F., Yazıcı Çelebi G. (2025). How Does Loneliness Affect Satisfaction with Life? What Is the Role of the Perception of God in This Interaction?. Front. Psychol..

[100] Martinez R.L., Regan A., Okabe-Miyamoto K., Lyubomirsky S. (2024). Only the Lonely: Learning, Use of Skills, and Sense of Meaning Buffer the Costs of Reduced Social Connection for Life Satisfaction. J. Posit. Psychol..

[101] Marttila E., Koivula A., Räsänen P. (2021). Does Excessive Social Media Use Decrease Subjective Well-Being? A Longitudinal Analysis of the Relationship between Problematic Use, Loneliness and Life Satisfaction. Telemat. Inf..

[102] Kupcewicz E., Mikla M., Kadučáková H., Grochans E. (2022). Loneliness and Satisfaction with Life among Nursing Students in Poland, Spain and Slovakia during the COVID-19 Pandemic. Int. J. Environ. Res. Public Health.

[103] Pilarska A., Suchańska A. (2021). A Shortened Version of the Indirect Self-Destructiveness Scale (ISDS-25). Psychiatr. Pol..

[104] Diener E., Emmons R.A., Larsen R.J., Griffin S. (1985). The Satisfaction with Life Scale. J. Pers. Assess..

[105] Juczyński Z. (2001). Narzędzia Pomiaru w Promocji i Psychologii Zdrowia. Skala Satysfakcji z Życia.

[106] Happ Z., Bodó-Varga Z., Bandi S.A., Kiss E.C., Nagy L., Csókási K. (2023). How Codependency Affects Dyadic Coping, Relationship Perception and Life Satisfaction. Curr. Psychol..

[107] De Jong Gierveld J., Van Tilburg T. (2006). A 6-Item Scale for Overall, Emotional, and Social Loneliness: Confirmatory Tests on Survey Data. Res. Aging.

[108] Humenny G., Grygiel P., Pokropek A. (2015). Poza ścisłą jedno- i wielowymiarowością. Struktura czynnikowa skali samotności de Jong Gierveld wśród dzieci. Modele Cech Ukrytych w Badaniach Edukacyjnych, Psychologii i Socjologii.

[109] Tucker M.C., Rodriguez C.M. (2014). Family Dysfunction and Social Isolation as Moderators between Stress and Child Physical Abuse Risk. J. Fam. Viol..

[110] Schober P., Boer C., Schwarte L.A. (2018). Correlation Coefficients: Appropriate Use and Interpretation. Anesth. Analg..

[111] Hayes A.F. (2018). Introduction to Mediation, Moderation, and Conditional Process Analysis: A Regression-Based Approach.

[112] Faul F., Erdfelder E., Lang A.G., Buchner A. (2007). G*Power 3: A Flexible Statistical Power Analysis Program for the Social, Behavioral, and Biomedical Sciences. Behav. Res. Methods.

[113] Richard F.D., Bond C.F., Stokes-Zoota J.J. (2003). One Hundred Years of Social Psychology Quantitatively Described. Rev. Gen. Psychol..

[114] Mishra P., Pandey C.M., Singh U., Gupta A., Sahu C., Keshri A. (2019). Descriptive Statistics and Normality Tests for Statistical Data. Ann. Card. Anaesth..

[115] Tsirigotis K. (2021). Emotional Intelligence, Indirect Self-Destructiveness and Gender. Psychiatr. Psychol. Klin..

[116] Bristow L.A., Afifi T.O., Salmon S., Katz L.Y. (2022). Risky Gambling Behaviors: Associations with Mental Health and a History of Adverse Childhood Experiences (ACEs). J. Gambl. Stud..

[117] Evans E., Hawton K., Rodham K. (2005). In What Ways Are Adolescents Who Engage in Self-Harm or Experience Thoughts of Self-Harm Different in Terms of Help-Seeking, Communication and Coping Strategies?. J. Adolesc..

[118] Troya M.I., Babatunde O., Polidano K., Bartlam B., McCloskey E., Dikomitis L., Chew-Graham C.A. (2019). Self-Harm in Older Adults: Systematic Review. Br. J. Psychiatry.

[119] Sadath A., Kavalidou K., McMahon E., Malone K., McLoughlin A. (2024). Associations between Humiliation, Shame, Self-Harm and Suicidality among Adolescents and Young Adults: A Systematic Review. PLoS ONE.

[120] Perlman D., Peplau L.A., Peplau L.A., Goldston S.E. (1984). Loneliness Research: Survey of Empirical Findings. Preventing the Harmful Consequences of Severe and Persistent Loneliness.

[121] Goodwin R., Cook O., Yung Y. (2001). Loneliness and Life Satisfaction among Three Cultural Groups. Pers. Relatsh..

[122] Swami V., Chamorro-Premuzic T., Sinniah D., Maniam T., Kannan K., Stanistreet D., Furnham A. (2007). General Health Mediates the Relationship between Loneliness, Life Satisfaction and Depression. Soc. Psychiatry Psychiatr. Epidemiol..

[123] Bozorgpour F., Salimi A. (2012). State Self-Esteem, Loneliness and Life Satisfaction. Procedia Soc. Behav. Sci..

[124] Tabassum S.A., Parveen A. (2024). Impact of Loneliness on Life Satisfaction among Students. Int. J. Multidiscip. Res..

[125] Bucher A., Neubauer A.B., Voss A., Oetzbach C. (2018). Together Is Better: Higher Committed Relationships Increase Life Satisfaction and Reduce Loneliness. J. Happiness Stud..

[126] Beck A.T., Weissman A., Lester D., Trexler L. (1974). The Measurement of Pessimism: The Hopelessness Scale. J. Consult. Clin. Psychol..

[127] Lee C.M., Cadigan J.M., Rhew I.C. (2020). Increases in Loneliness among Young Adults during the COVID-19 Pandemic and Association with Increases in Mental Health Problems. J. Adolesc. Health.

[128] Riddle J. (1991). The Dysfunctional Family: Cause and Effect. J. Community Psychol..

[129] Kasar K.S., Karaman E. (2021). Life in Lockdown: Social Isolation, Loneliness and Quality of Life in the Elderly during the COVID-19 Pandemic—A Scoping Review. Geriatr. Nurs..

[130] Funder D.C., Ozer D.J. (2019). Evaluating Effect Size in Psychological Research: Sense and Nonsense. Adv. Methods Pract. Psychol. Sci..

[131] Kenny D.A., Judd C.M. (2014). Power Anomalies in Testing Mediation. Psychol. Sci..

[132] Norman R.E., Byambaa M., De R., Butchart A., Scott J., Vos T. (2012). The Long-Term Health Consequences of Child Physical Abuse, Emotional Abuse, and Neglect: A Systematic Review and Meta-Analysis. PLoS Med..

[133] Kang W., Malvaso A., Whelan E. (2023). Asthma Moderates the Association between the Big Five Personality Traits and Life Satisfaction. Healthcare.

[134] Lahey B.B. (2009). Public Health Significance of Neuroticism. Am. Psychol..

[135] O’Connor R. (2021). When It Is Darkest: Why People Die by Suicide and What We Can Do to Prevent It.

[136] Mohamad M.A., Leong Bin Abdullah M.F.I., Shari N.I. (2024). Similarities and Differences in the Prevalence and Risk Factors of Suicidal Behavior between Caregivers and People with Dementia: A Systematic Review. BMC Geriatr..

